# Loss of ezrin expression reduced the susceptibility to the glomerular injury in mice

**DOI:** 10.1038/s41598-018-22846-0

**Published:** 2018-03-14

**Authors:** Ryo Hatano, Ai Takeda, Yukiko Abe, Kotoku Kawaguchi, Itsuro Kazama, Mitsunobu Matsubara, Shinji Asano

**Affiliations:** 1 Department of Molecular Physiology, College of Pharmaceutical Sciences, Ritsumeikan University, Kusatsu, Shiga Japan; 2 Department of Physiology, Tohoku University Graduate School of Medicine, Sendai, Miyagi Japan; 3 Division of Molecular Medicine, Center for Translational and Advanced Animal Research, Tohoku University Graduate School of Medicine, Sendai, Miyagi Japan

## Abstract

Ezrin is highly expressed in glomerular podocytes and is reported to form a multi-protein complex with scaffold protein Na^+^/H^+^ exchanger regulatory factor 2 (NHERF2) and podocalyxin, a major sialoprotein. Podocalyxin-knockout mice died within 24 h of birth with anuric renal failure, whereas NHERF2-knockout mice show no apparent changes in the glomerular functions. However, the physiological roles of ezrin in glomerular podocytes remain unclear. Here, we investigated the importance of ezrin in the regulation of glomerular podocyte function using ezrin-knockdown mice (*Vil2*^*kd/kd*^). The *Vil2*^*kd/kd*^ mice did not exhibit apparent glomerular dysfunction, morphological defects or abnormal localisation of podocalyxin and NHERF2 in podocytes. Thus, we investigated the influence of ezrin defects on Rho-GTPase activity, as ezrin interacts with the Rho-GTPase dissociation inhibitor (Rho-GDI), which plays a key role in the regulation of podocyte actin organisation. In *Vil2*^*kd/kd*^ glomeruli, Rac1 activity was significantly reduced compared to wildtype (WT) glomeruli at baseline. Furthermore, *Vil2*^*kd/kd*^ mice showed reduced susceptibility to glomerular injury. In WT glomeruli, Rac1 activity was enhanced in nephrotic conditions, but remained at baseline levels in *Vil2*^*kd/kd*^ glomeruli, suggesting that loss of ezrin protects podocytes from injury-induced morphological changes by suppressing Rac1 activation.

## Introduction

Ezrin is a member of the ezrin-radixin-moesin (ERM) family of proteins, which have divergent roles in the regulation of cellular function^1^. Ezrin is mainly expressed in epithelial tissues, including kidneys and gastrointestinal tissues, where it plays diverse physiological functions^2–4^. In kidneys, intense expression of ezrin is observed in proximal tubules and glomerular podocytes^5^. In proximal tubules, ezrin plays important roles in tubular solute reabsorption via the regulation of apical membrane localisation of several transporters^2^. The interaction between ezrin and Na^+^/H^+^ exchanger regulatory factor 1 (NHERF1), which possesses PDZ domains, enables transporters possessing a PDZ binding motif mainly located at the cytoplasmic carboxyl-terminus to localise at apical membranes^2,6^. We previously reported that ezrin-knockdown (*Vil2*^*kd/kd*^) mice exhibited reduced phosphate reabsorption in their proximal tubules, resulting in hypophosphatemia^2^. However, the physiological function of ezrin in glomerular podocytes remains unclear. Orlando *et al*.^7^ reported that ezrin localises at the luminal membrane of podocytes and forms a complex with podocalyxin, which is one of the sialoproteins, and that this interaction was intermediated by NHERF2. Podocalyxin-knockout mice revealed perinatal lethality due to abnormal foot processes and slit diaphragm formation in the glomerular podocytes^8^. However, the NHERF2-knockout mice did not show an apparent renal phenotype^9^. On the contrary, Wegner *et al*. reported that CLIC5A, a member of the chloride intracellular channel, plays an important role in podocyte foot process formation via macromolecular complex formation with ezrin and podocalyxin; furthermore, CLIC5-knockout mice exhibited susceptibility to glomerular injury, possibly due to the reduction in ezrin expression^10^.

Ezrin functions not only as a scaffold for transmembrane proteins, but also as a regulator of the small GTPase Rho. Ezrin is capable of interacting with the Rho GDP dissociation inhibitor (Rho-GDI), and indirectly activates Rho-GTPase by promoting the dissociation of GDP-bound Rho-GTPase from Rho-GDI^11^. The essential roles of Rho-GTPase in actin organisation in glomerular podocytes have been recently discussed^12,13^. Three major kinds of Rho-GTPases—namely RhoA, Cdc42 and Rac1—play important roles in podocyte actin organisation^12,13^. Disturbances in the balance of these GTPases can lead to actin rearrangement and morphological changes, such as regression of foot processes in podocytes, resulting in severe proteinuria and glomerular injury. Shibata *et al*. previously reported that Rho-GDIα knockout caused severe proteinuria by hyperactivation of Rac1 in podocytes^14^. Thus, ezrin might play important roles in actin organisation via the regulation of Rho-GTPase activity in podocytes. In the present study, we examined the physiological function of ezrin in glomerular podocytes using *Vil2*^*kd/kd*^ mice.

## Results

### No apparent morphological defects in *Vil2*^*kd/kd*^ mouse glomeruli

**Figure 1:**
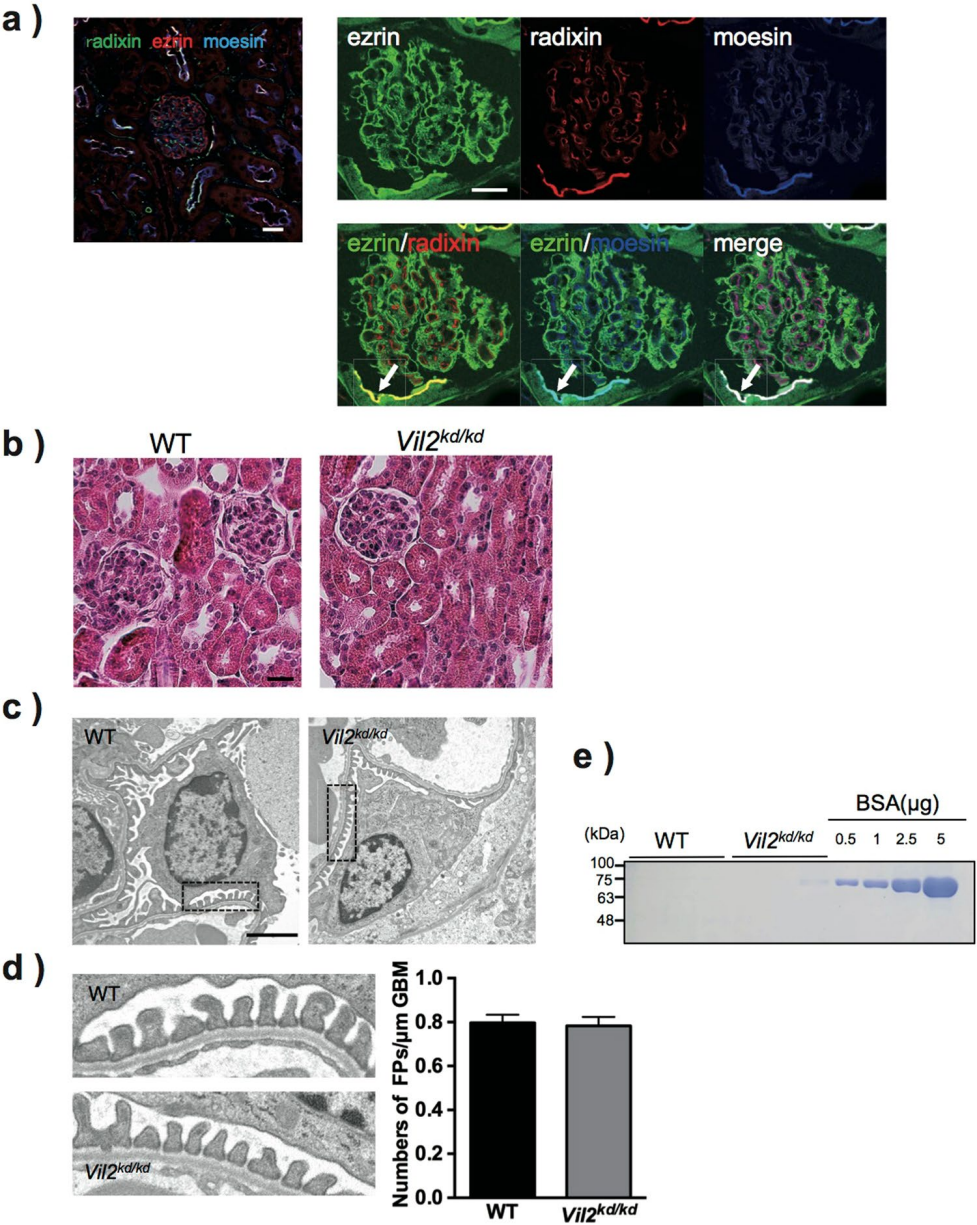
Distributions of ERM proteins in mouse glomeruli and histological analysis of *Vil2*^*kd/kd*^ mouse glomeruli. In WT mouse kidneys, glomerular localisation of ERM proteins was investigated by immunofluorescence analysis. All three proteins were commonly detected in the apical membrane of proximal tubules, but showed different localisation in glomeruli (**a**) left panel, green: radixin, red: ezrin and blue: moesin). In glomeruli, ezrin was exclusively detected in podocytes; radixin and moesin were detected in endothelial cells, but not podocytes (**a**) right three panels). Radixin and moesin co-localised with ezrin in the apical membranes of the proximal tubules (*Arrow*). Scale bar: 20 μm. There were no morphological abnormalities in the *Vil2*^*kd/kd*^ glomeruli observed by H&E staining (Scale bar: 25 μm) (**b**) or electron microscopic analysis (magnification × 3,610, scale bar: 2 μm) (**c**). Areas enclosed by dotted lines are magnified, and (magnification × 19,000) were shown in (**d**) and the number of foot processes/μm of glomerular basement membrane (GBM) was counted (**d**) right graph). Spot urine was separated by SDS-PAGE and stained with CBB. As a positive control, BSA (0.5, 1, 2.5 and 5 μg) was loaded. Apparent urinary albumin leakage was not observed in *Vil2*^*kd/kd*^ mouse urine (**e**).

Ezrin was highly expressed in the glomerular podocytes and apical membranes of the proximal tubules in the kidneys Fig. 1a. Radixin and moesin were not observed in the podocytes, although they co-localised with ezrin in the apical membranes of the proximal tubules Fig. 1a. Ezrin was not co-localised with CD34, an endothelial cell marker, although moesin was partially merged with CD34 Supp. Fig. S1a,b. Moesin was not merged with desmin, a mesangial marker Supp. Fig. S1c. Histological analysis of mouse kidneys was performed to determine the effect of ezrin-knockdown on glomerular morphology. H & E staining showed no apparent difference in glomerular morphologies between WT and *Vil2*^*kd/kd*^ mice at the basal condition Fig. 1b. Next, we performed electron microscopic analysis of glomerular structures for more detailed structural examination. However, no apparent morphological change was observed in the glomerular podocyte foot process in *Vil2*^*kd/kd*^ mice at the basal condition Fig. 1c. There was also no difference in the number of foot processes per micrometre of glomerular basal membrane (GBM) Fig. 1d. We then performed biochemical analysis using urine and plasma samples. There was no significant difference in urinary albumin excretion, as shown by the urinary albumin/creatinine ratio (ACR), between WT and *Vil2*^*kd/kd*^ mice (WT: 0.31 ± 0.11, *Vil2*^*kd/kd*^: 0.21 ± 0.06 μg/mg creatinine). CBB staining for electrophoresed urine samples showed no obvious bands around ~60 kDa (as predicted for albumin) in either WT- or *Vil2*^*kd/kd*^-mouse urine Fig. 1e, suggesting no leakage of plasma albumin into the urine of *Vil2*^*kd/kd*^ mice, which is in concordance with the results of our histological analysis.

### Subcellular localisation of ezrin-associated proteins in WT and *Vil2*^*kd/kd*^ mouse podocytes

**Figure 2:**
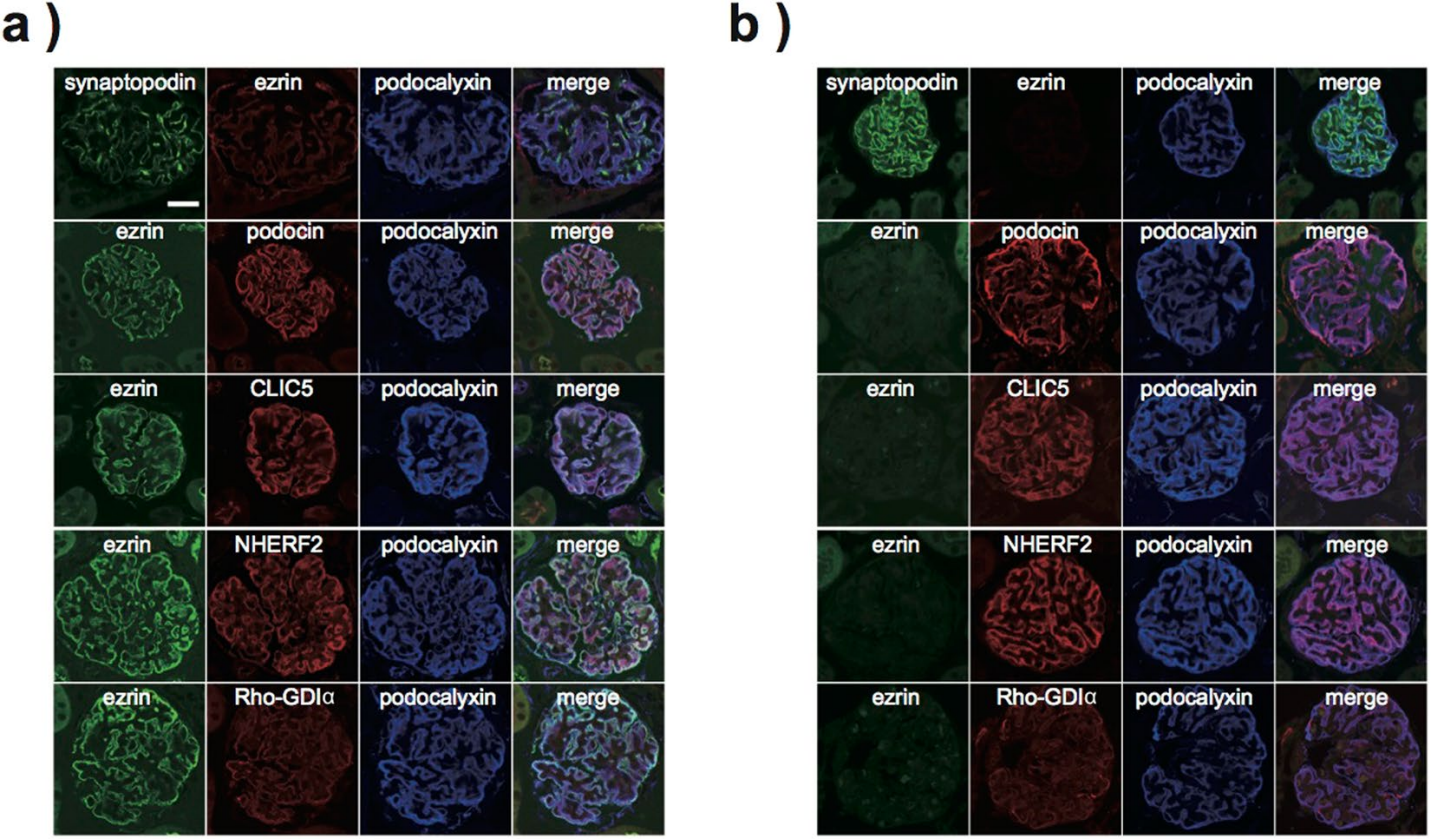
Immunofluorescence analysis for proteins expressed in glomerular podocytes. Immunolocalisation of ezrin, other related proteins (CLIC5, NHERF2, Rho-GDIα and podocalyxin), and marker proteins (synaptopodin and podocin) was investigated in (**a**) WT and (**b**) *Vil2*^*kd/kd*^ mouse glomeruli (Scale bar: 20 μm).

We also investigated the subcellular localisation of ezrin-interacting proteins including NHERF2 and podocalyxin by coimmunostaining with podocyte marker proteins (synaptopodin and podocin). Ezrin was mainly detected in the apical membrane of podocytes in WT glomeruli and did not merge well with synaptopodin and podocin, which were mainly detected in the foot processes close to GBM Fig. 2a. On the contrary, ezrin co-localised with podocalyxin, NHERF2 and CLIC5 in the apical membrane of podocytes, as previously reported, whereas CLIC5 positive staining was found in glomerular capillaries Fig. 2a. In *Vil2*^*kd/kd*^ podocytes, ezrin expression was dramatically reduced compared with WT podocytes Fig. 2a,b. There was no apparent change in the localisation of radixin and moesin Supp. Fig. S1d, e. There was also no apparent change in the foot process localisation of both synaptopodin and podocin Fig. 2b, which is in agreement with the results of the histological analyses showing no morphological abnormality in *Vil2*^*kd/kd*^ podocytes. Furthermore, podocalyxin, NHERF2 and CLIC5 were detected in the apical membrane of podocytes in *Vil2*^*kd/kd*^ mice, suggesting that ezrin is not required for the appropriate apical membrane localisation of these proteins in podocytes. In addition, the expression level and subcellular localisation of Rho-GDIα were not altered in *Vil2*^*kd/kd*^ podocytes compared with WT podocytes Fig. 2a,b.

### Expression levels of ezrin-associated proteins in isolated glomeruli

**Figure 3:**
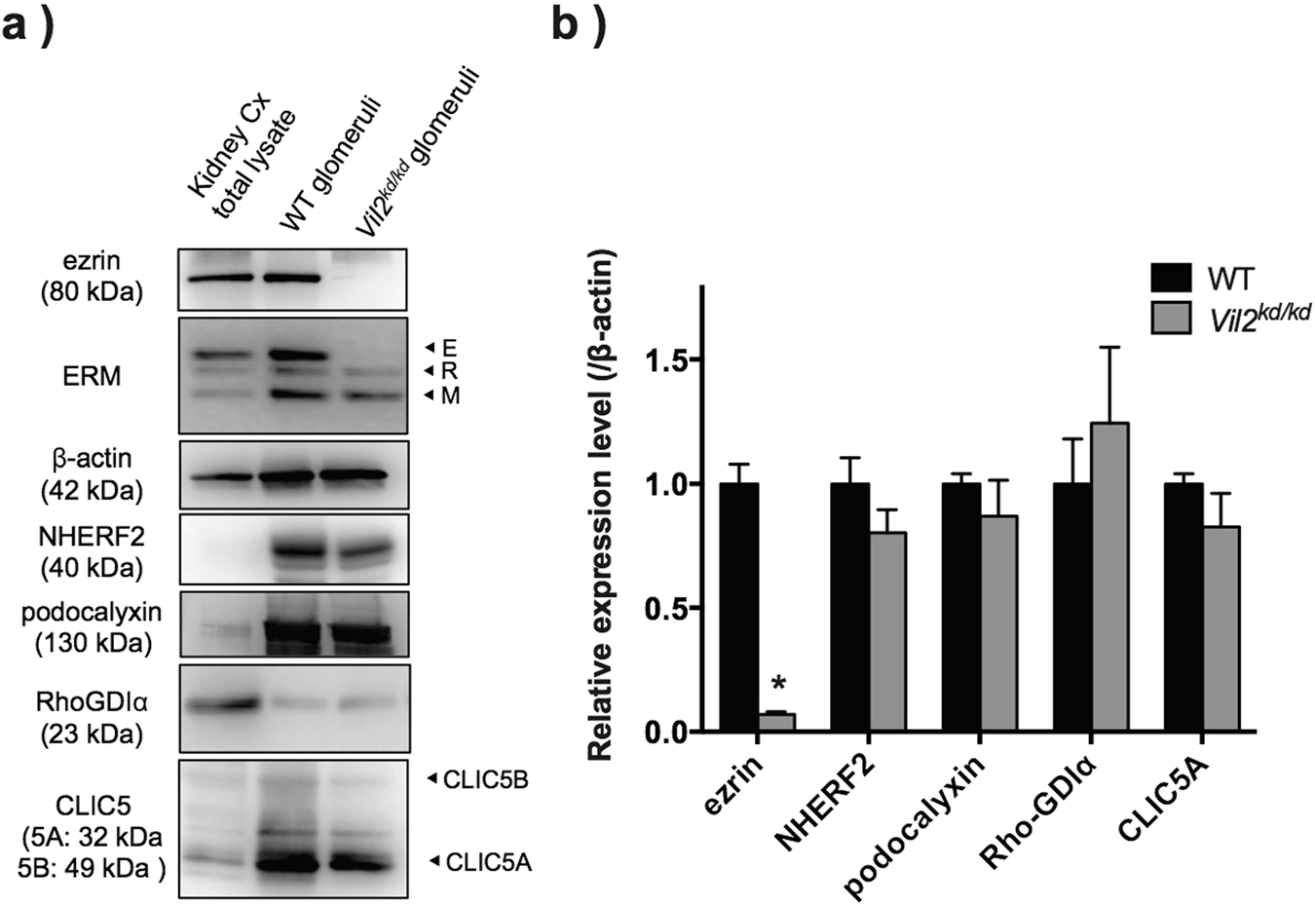
Immunoblot analysis of glomerular proteins in isolated glomeruli from WT and *Vil2*^*kd/kd*^ mice. (**a**) Glomerular protein expression levels were investigated by immunoblotting. As a control, total kidney cortex lysate from WT mouse was used. (**b**) Densitometric analysis was performed (n = 3–5, respectively). ^*^P < 0.05, vs. WT.

Protein abundance was investigated by immunoblotting using isolated glomeruli from both WT and *Vil2*^*kd/kd*^ mice. Glomeruli were isolated by the magnetic bead-based isolation technique as previously described^15^. Among the ERM proteins, ezrin and moesin were abundantly detected in the isolated glomeruli from WT mice. Ezrin expression levels were dramatically reduced in *Vil2*^*kd/kd*^ mouse glomeruli, consistent with immunostaining Fig. 3a,b. Compensatory up-regulation of radixin and moesin expression was not observed in *Vil2*^*kd/kd*^ mouse glomeruli Fig. 3a,b. Podocalyxin expression levels were similar between WT and *Vil2*^*kd/kd*^ mice, whereas NHERF2 expression levels were slightly—but not significantly—reduced in *Vil2*^*kd/kd*^ mice. CLIC5A expression levels were comparable between WT and *Vil2*^*kd/kd*^ mice Fig. 3a,b. Expression levels of Rho-GDIα were also unaltered in *Vil2*^*kd/kd*^ mice compared with WT mice.

### Investigation of susceptibility to adriamycin and lipopolysaccharide-induced nephropathy

**Figure 4:**
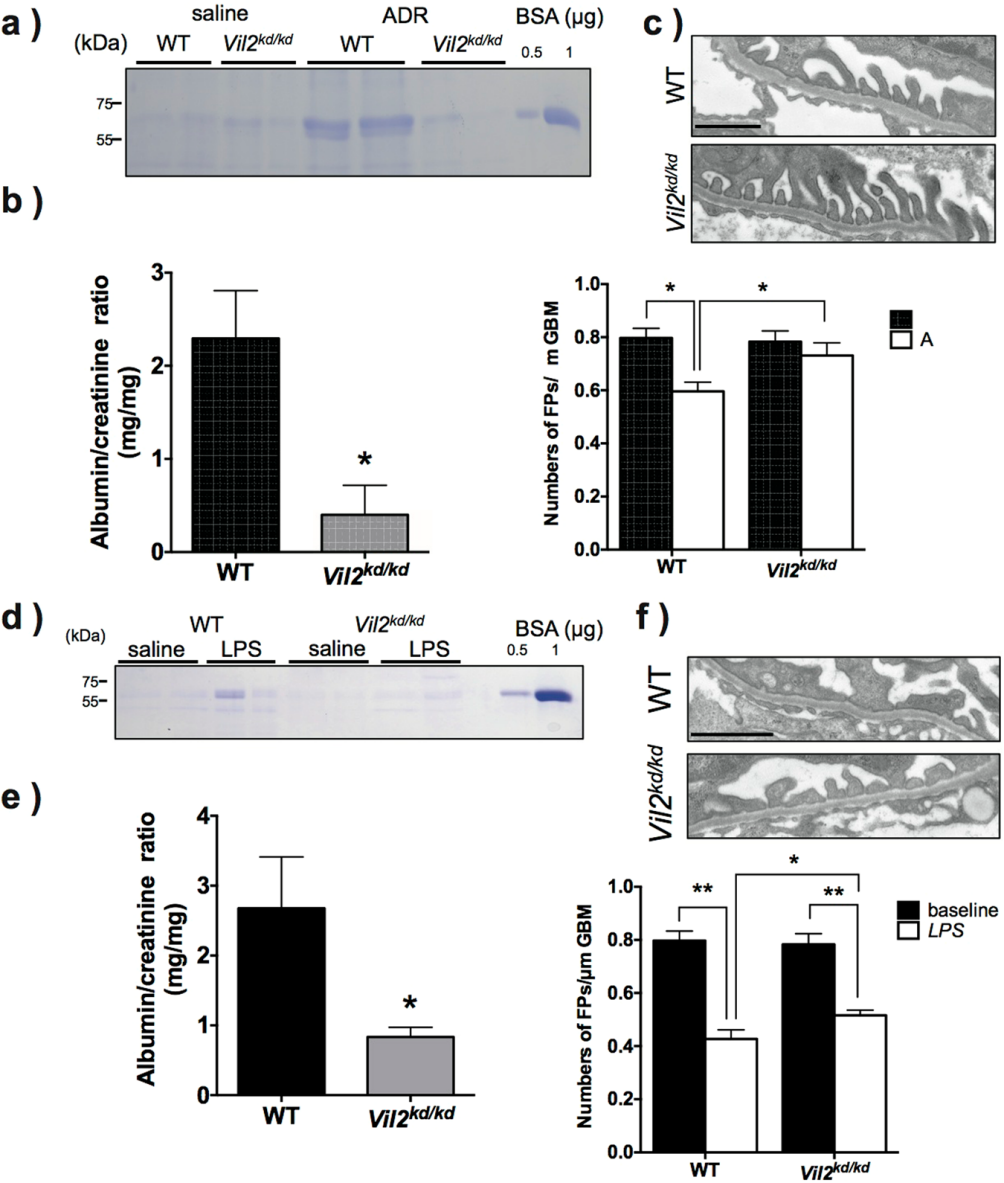
Morphological changes in podocytes and functional changes in ADR- or LPS-induced glomerulopathy. Adriamycin (50 mg/kg) was administrated intravenously. After 7 days, spot urine was collected, separated by SDS-PAGE and stained with CBB. As a positive control, BSA (0.5 and 1 μg) was loaded. Urinary albumin excretion was increased in WT mice but not *Vil2*^*kd/kd*^ mice (WT: n = 7, *Vil2*^*kd/kd*^: n = 7) (**a**). Urinary albumin and creatinine concentrations were measured and ACR (mg albumin/mg creatinine) was calculated (**b**). Electron microscopic analysis was performed in these mice (magnification × 19,000, Scale bar: 1 μm). (**c**) Number of foot processes/μm of GBM was measured. Foot process effacement was significantly progressed in WT mice, but not *Vil2*^*kd/kd*^ mice. LPS (200 μg) was administrated intraperitoneally. After 24 h, spot urine was collected, separated by SDS-PAGE, and stained with CBB (**d**). As a positive control, BSA (0.5 and 1 μg) was loaded. Urinary albumin and creatinine concentrations were measured and ACR(mg albumin/mg creatinine) was calculated (WT: n = 9, *Vil2*^*kd/kd*^: n = 9) (**e**). Electron microscopic analysis was performed in these mice (magnification × 19,000, Scale bar: 1 μm). (**f**) Number of foot processes/μm of GBM was measured.

To determine susceptibility to glomerular injury we adopted two nephrotic models: Adriamycin (ADR)-induced and lipopolysaccharide (LPS)-induced glomerular injury. Seven days after ADR administration, WT mice exhibited enhanced urinary albumin excretion, but *Vil2*^*kd/kd*^ mice did not show an apparent increase in urinary albumin content Fig. 4a,b. Electron microscopy showed increased frequency of foot process effacement in ADR-treated WT mice compared with non-treated WT mice Fig. 4c. Interestingly, the increase in foot process effacement was not observed in *Vil2*^*kd/kd*^ mice Fig. 4c. We also examined LPS-induced nephrotic condition using these mice. In LPS-induced glomerular injury, WT mice showed an apparent urinary leak of albumin 24 h after LPS injection Fig. 4d,e. *Vil2*^*kd/kd*^ mice also showed a significant increase in urinary albumin excretion, although it was milder compared to WT mice Fig. 4d,e. Using electron microscopy, we observed severe foot process effacement in LPS-treated WT mice, but the foot process effacement was relatively moderate in LPS-treated *Vil2*^*kd/kd*^ mice Fig. 4f. These results suggest that defective expression of ezrin is beneficial for protection from podocyte injury.

### Investigation of Rho-GTPase activity in isolated glomeruli

**Figure 5:**
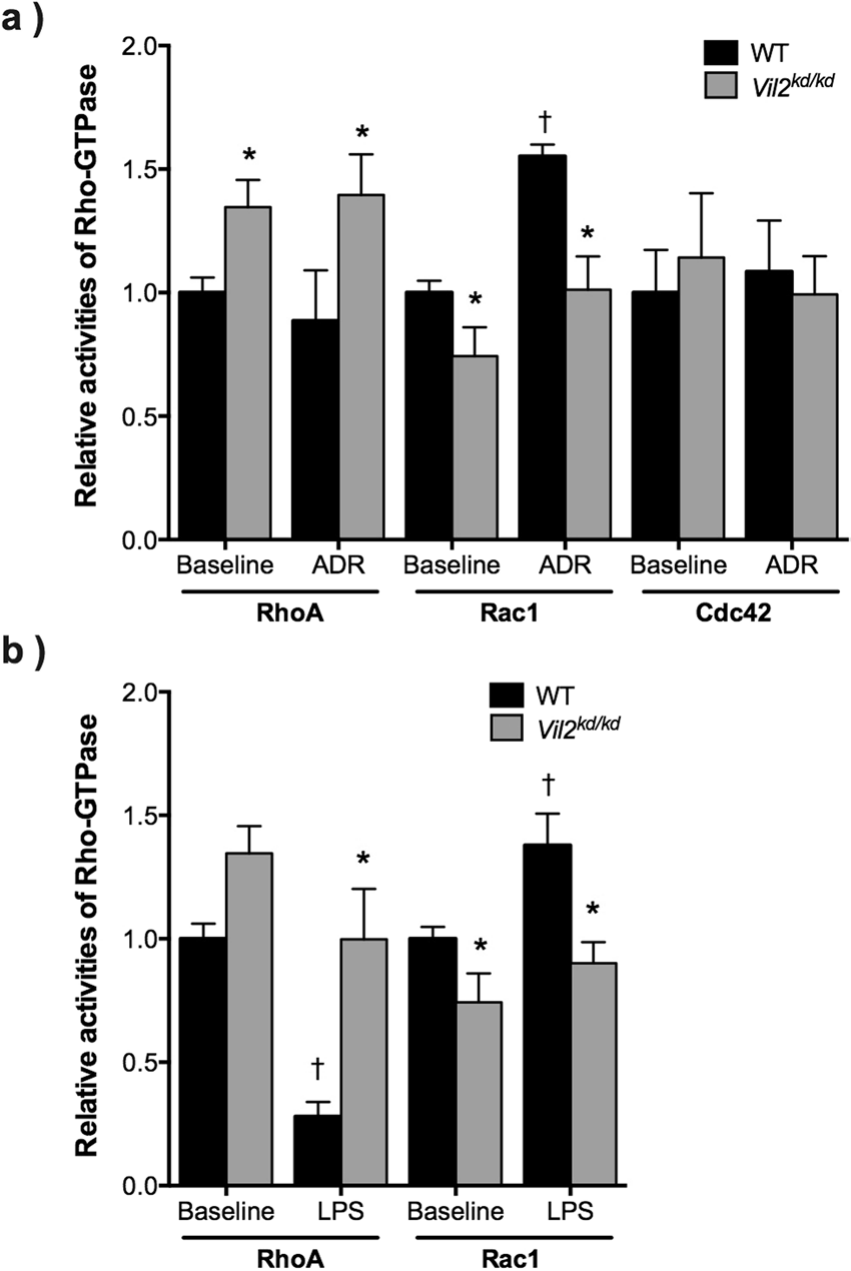
Rho-GTPase activity assay using isolated glomeruli. Rho-GTPase activity—including RhoA, Rac1 and Cdc42—was measured by an ELISA-based Rho-G-LISA assay. Isolated glomeruli from both untreated and ADR-treated WT and *Vil2*^*kd/kd*^ mice were used in this study (n = 5–11) (**a**). Isolated glomeruli from both untreated and LPS-treated WT and *Vil2*^*kd/kd*^ mice were used in this study (n = 5–8 (**b**). ^*^P < 0.05, *vs*. WT at the same condition. ^†^p < 0.05, vs. baseline for the same genotype.

In podocytes, Rho-GTPase plays a key role in the progression of podocyte injuries, as mentioned above. Three members of Rho-GTPase—namely RhoA, Cdc42 and Rac1—have different roles in the maintenance of actin cytoskeletal dynamics in podocyte foot processes. Therefore, we investigated Rho-GTPase activity in isolated glomeruli from WT and *Vil2*^*kd/kd*^ mice. At the basal condition, RhoA activity in *Vil2*^*kd/kd*^ glomeruli was significantly higher than in WT glomeruli, whereas Rac1 activity was significantly lower in *Vil2*^*kd/kd*^ glomeruli Fig. 5a. On the contrary, Cdc42 activity in *Vil2*^*kd/kd*^ glomeruli was comparable to that in WT glomeruli Fig. 5a. We further investigated Rho-GTPase activity in ADR- and LPS-injected mice. In ADR-injected mice, RhoA activity in *Vil2*^*kd/kd*^ glomeruli was maintained at a significantly higher level compared to WT glomeruli. On the contrary, Rac1 activity in *Vil2*^*kd/kd*^ glomeruli was significantly lower than in WT glomeruli Fig. 5a. In LPS-injected mice, RhoA activity was significantly reduced in WT mice Fig. 5b, but maintained at the baseline level in *Vil2*^*kd/kd*^ mice Fig. 5b. Rac1 activity was significantly increased in LPS-injected WT mice, whereas it was maintained at the baseline level in *Vil2*^*kd/kd*^ mice Fig. 5b. These results suggest that both up-regulated RhoA activity and down-regulated Rac1 activity in *Vil2*^*kd/kd*^ glomeruli contributed to keeping podocytes in a static condition when the glomerular podocytes were injured by ADR or LPS.

### *In vitro* analysis using cultured immortalised podocytes

**Figure 6:**
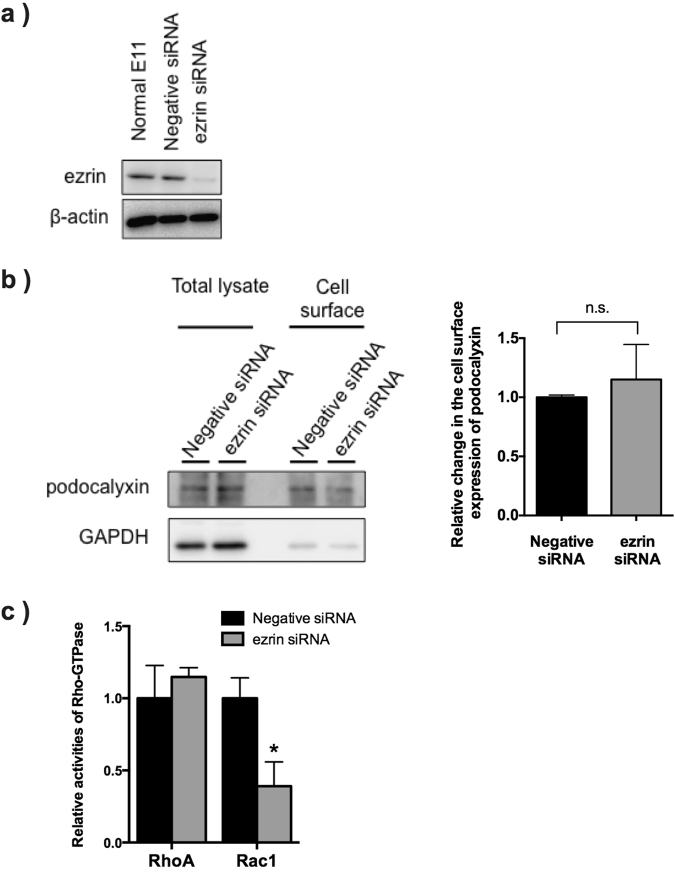
*In vitro* analysis of surface expression of podocalyxin and Rho-GTPase activity using a podocyte cell line. ERM protein expression levels in E11 cells were investigated by immunoblotting. Knockdown of ezrin was performed by siRNA. The siRNA targeting ezrin gene effectively down-regulated expression of ezrin in E11 cells (**a**). A surface biotinylation assay was performed using negative control siRNA- and ezrin siRNA-treated E11 cells. Surface expression of podocalyxin was examined and no contamination of cytosolic proteins was identified by immunoblotting for GAPDH. Densitometry analysis was performed (n = 5 for each group) (**b**). RhoA and Rac1 activity in siRNA-treated E11 cells was investigated by Rho-GTPase activity assay (n = 5 for each group). ^*^P < 0.05, vs. Negative siRNA-treated E11 cells (**c**).

**Figure 7:**
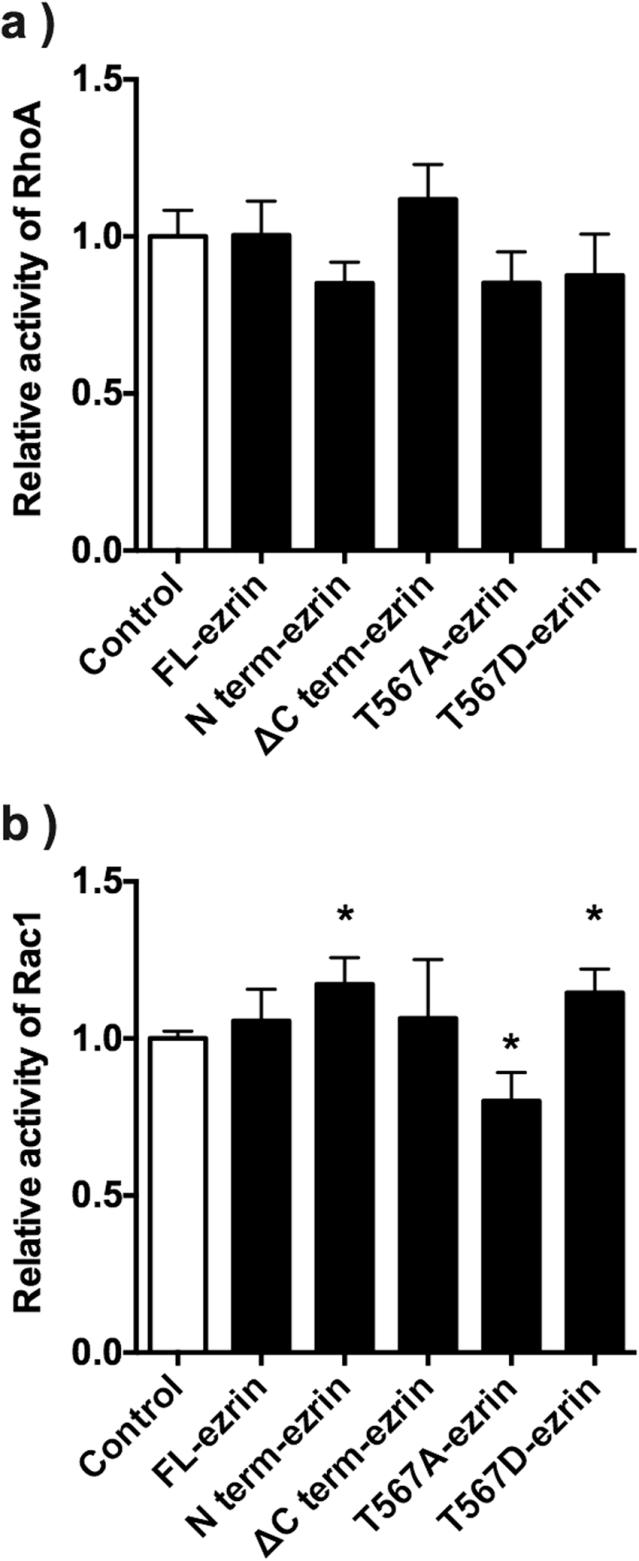
RhoA and Rac1 activity in E11 cells transfected with ezrin mutants. E11 cells were transfected with different kinds of ezrin mutants (Full length ezrin: FL-ezrin; N-terminal (FERM) domain (aa:1-310): N term-ezrin; C-terminal deletion mutant (aa: 1-533): ΔC term-ezrin; constitutively inactive ezrin: T567A-ezrin; and constitutively active ezrin: T567D-ezrin). RhoA (**a**) and Rac1 (**b**) activity was measured in these E11 cells. ^*^P < 0.05, vs. control. (n = 3–5).

A cultured immortalised podocyte cell line, E11 cells, were used in this study. E11 cells abundantly express ezrin Fig. 6a. Thus, we performed knockdown of ezrin in E11 cells using siRNA to investigate whether loss of ezrin affects membrane localisation of podocalyxin or Rho-GTPase activity in podocytes. Ezrin expression was effectively down-regulated by siRNA Fig. 6a. There was no significant difference in podocalyxin expression levels between control E11 and ezrin-knockdown E11 cells Fig. 6b: Total lysate. When we performed a cell surface biotinylation assay using these cells, the abundance of podocalyxin at membrane surface was not altered Fig. 6b: Cell surface, suggesting that loss of ezrin did not affect membrane localisation of podocalyxin in the glomerular podocytes. Furthermore, we performed a Rho-GTPase activity assay using these cells. In the ezrin-knockdown E11 cells, Rac1 activity was significantly decreased, whereas RhoA activity was comparable between control and ezrin-knockdown E11 cells Fig. 6c. To investigate whether ezrin activity influences Rho-GTPase activity in podocytes, we transfected several kinds of ezrin mutant genes in E11 cells. We then investigated Rho-GTPase activity in E11 cells after transfection of constitutively active (T567D) ezrin (T567D-ezrin), constitutively inactive (T567A) ezrin (T567A-ezrin), ezrin lacking an actin-binding domain at the C-terminus (1–533 aa: ΔCt-ezrin), full-length ezrin (FL-ezrin) and the N-terminal (FERM: 4.1-ezrin-radixin-moesin) domain of ezrin (1–310aa: N term-ezrin). All these proteins were conjugated with a flag-tag at the N-terminal. In all transfected cells, RhoA activity was not significantly altered Fig. 7a. However, Rac1 activity was significantly increased in both T567D-ezrin and N term-ezrin transfected E11 cells and significantly reduced in T567A-ezrin transfected E11 cells Fig. 7b. In FL-ezrin and ΔCt-ezrin transfected E11 cells, Rac1 activity was not significantly altered Fig. 7b.

### Morphological analysis of glomerular podocytes transfected with ezrin mutants

**Figure 8:**
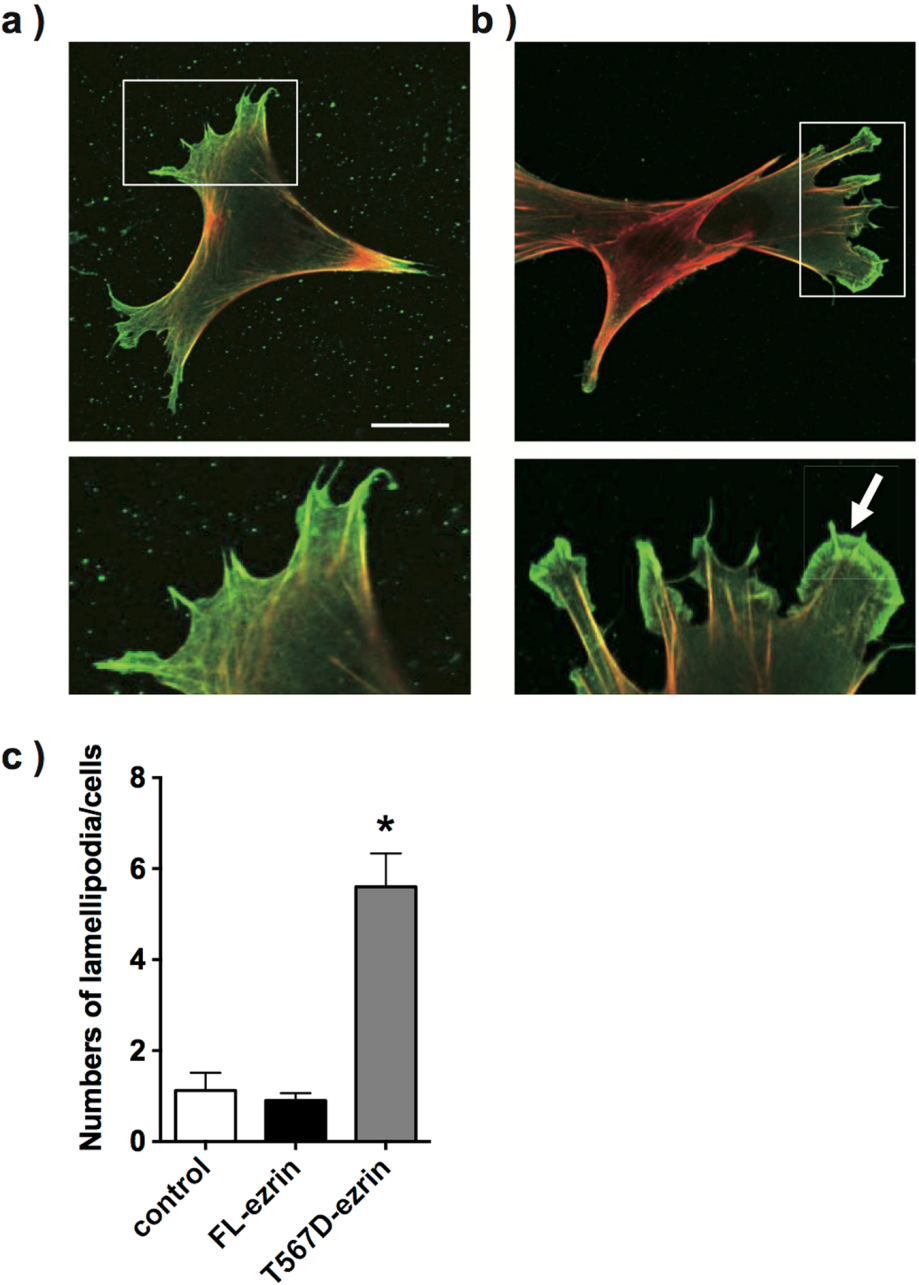
Morphological analysis of FL-ezrin and T567D-ezrin transfected E11 cells. E11 cells transfected with FL-ezrin (**a**) and T567D-ezrin (**b**) were double-stained with an anti-flag antibody (green) and rhodamine-phalloidin (red). Both FL-ezrin and T567D-ezrin were detected at the cellular cortex. Some protrusions were observed in FL-ezrin-transfected E11 cells (**a**), while enhanced membrane ruffling was observed in T567D-ezrin transfected E11 cells (white arrow) (**b**). The number of lamellipodia per cell was counted. Lamellipodia were defined as 3- to 10-μm regions located at the cell periphery, as previously described by Raucher *et al*.

Immunofluorescence analysis was performed on FL-ezrin and T567D-ezrin transfected E11 cells. Cells were stained with rhodamine-phalloidin and an anti-flag antibody. Transfected flag-tagged ezrin was observed at the cell surface Fig. 8a,b. Some protrusions were observed in the FL-ezrin transfected E11 cells Fig. 8a. By contrast, in T567D-ezrin transfected E11 cells, enhanced membrane ruffling was observed Fig. 8b, consistent with increased Rac1 activity Fig. 7b. These results suggest that ezrin regulates the cellular morphology of podocytes through the regulation of Rac1 activity.

## Discussion

Ezrin is an actin-binding protein that is highly expressed in glomerular podocytes and proximal tubules in kidneys. Since ezrin is capable of interacting with several proteins related to the maintenance of podocyte morphology, ezrin is predicted to play important physiological roles in the regulation of glomerular function. However, in our present study, loss of ezrin did not appear to affect glomerular function or podocyte morphology in mice at the basal condition. Furthermore, we showed that *Vil2*^*kd/kd*^ mice exhibited a protective phenotype against glomerular injury by the suppression of Rac1 activation in glomerular podocytes.

In glomeruli, ezrin is believed to play an important role as a scaffold for podocalyxin^7^. Takeda *et al*. reported that the disruption of multi-protein complex formation was correlated with a severe proteinuric phenotype in nephrotic model rats^16^, yet it remains unclear whether disruption of complex formation directly caused glomerular injury. Wegner *et al*. identified ezrin as a CLIC5A-binding protein and reported that decreased ezrin expression is implicated in glomerular dysfunction in CLIC5A-knockout mice, since ezrin was down-regulated in the CLIC5A^−/−^ podocytes^10^. Their hypothesis was also based on the report that disruption of multi-protein complex formation caused podocyte injury. However, no defects in glomerular function were observed in either NHERF2-knockout mice^9^ or ezrin-knockdown mice in the present study. Therefore, we must carefully investigate the physiological importance of multi-protein complex formation. Our data, and previous data in NHERF2-knockout mice^9^, suggest that multi-protein complex formation is not necessary for appropriate apical surface localisation of podocalyxin in podocytes, and that ezrin has different functional roles in podocytes, not only scaffolding of proteins. Madin-Darby canine kidney (MDCK) cells, an established epithelial cell line, are often used for the analysis of complex formation^7,17,18^; perhaps complex formation is physiologically important in polarised epithelial cells.

Ezrin also has an important function as a regulator of actin organisation, as described above. Recently, it has been suggested that the three Rho-GTPases—namely RhoA, Rac1 and Cdc42—orchestrate actin organisation in glomerular podocytes^12,13^. Disturbance in the balance between these Rho-GTPases has been implicated in the progression of glomerular podocyte injury as a result of fusion of foot processes^12,13,19^. RhoA and Rac1/Cdc42 antagonistically regulate each other. RhoA stabilises podocyte foot processes and protects podocytes from effacement, whereas Rac1 and Cdc42 promote foot process motility and the development of foot process effacement^19^. An increase in Rac1 activity and decrease in RhoA activity are likely to be associated with the severity of glomerular injury in these models. In ADR-treated WT mice, Rac1 activity significantly increased, but RhoA activity was not significantly altered. In LPS-treated WT mice, which showed more severe glomerular injury, both activation of Rac1 and suppression of RhoA activity were observed. Therefore, activation of Rac1 might primarily occur after glomerular injury. In *Vil2*^*kd/kd*^ glomeruli, Rac1 activity significantly decreased, possibly due to the defective interaction between ezrin and Rho-GDIα. Therefore, the *Vil2*^*kd/kd*^ podocytes should be protected from Rac1-mediated morphological changes in these glomerular injury models. Shibata *et al*. reported that Rho-GDIα plays an important role in the foot process structure^14^. In Rho-GDIα-knockout mice, Rac1 activity was abnormally activated, and thus foot process effacement was induced. On the contrary, Casaletto *et al*. reported that RhoA activity was increased in the enterocytes of ezrin-knockout mice^4^, although it was not clear whether this was due to disrupted interactions between ezrin and Rho-GDI. RhoA activity after ADR and LPS treatment was significantly higher in *Vil2*^*kd/kd*^ glomeruli than in WT glomeruli. The change in RhoA activity might be secondary to the reduction in Rac1 activity since RhoA and Rac1 are mutually antagonistic^20,21^. Thus, RhoA activity should be enhanced due to the reduced Rac1 activity in *Vil2*^*kd/kd*^ podocytes, which may prevent any increase in motility induced by glomerular injury. Furthermore, our study suggests that phosphorylation of ezrin was enhanced in WT glomeruli after ADR and LPS treatment Supp. Fig. S2a,b. Hsu *et al*. similarly reported that phosphorylation of ERM proteins was enhanced after angiotensin II-induced podocyte injury^22^, suggesting that activation of ezrin is associated with the progression of glomerular injury Fig. 8.

We examined *in vitro* experiments using transfection of siRNA or ezrin mutants. Suppression of ezrin expression and transfection of the constitutively inactive mutant of ezrin (T567A ezrin) resulted in a reduction in Rac1 activity. However, Rac1 activity in both the constitutively active mutant of ezrin (T567D ezrin)- and N-terminal FERM domain (N term-ezrin)-transfected E11 cells was significantly enhanced. These results are consistent with a previous study by Takahashi *et al*.^11^ reporting that the N-terminal domain of ERM proteins activated Rho-GTPase activity. Taken together, these results suggest that the interaction between ezrin and Rho-GDIα is important for the regulation of Rho-GTPase activity in glomerular podocytes. In E11 cells, RhoA activity was not significantly altered by knockdown of ezrin or overexpression of ezrin mutants, suggesting that ezrin plays more important roles in the regulation of Rac1 activity through its interaction with Rho-GDIα. Furthermore, overexpression of FL-ezrin did not affect Rac1 activity or cell morphology, but the constitutively active form of ezrin (T567D-ezrin) activated Rac1 and lamellipodia formation. These results support that the phosphorylated form of ezrin plays important roles in the regulation of podocyte morphology through Rac1 activation.

Ezrin is also expressed in the proximal tubules of kidneys. As we previously reported, *Vil2*^*kd/kd*^ mice showed impaired phosphate reabsorption in their proximal tubules. Therefore, loss of ezrin might affect proximal tubular reabsorption of proteins filtered through glomeruli. We observed that levels of urinary β_2_-microglobulin, a low-molecular-weight protein, which passes through the glomerular filtration barrier and is reabsorbed by the proximal tubules, were slightly increased in *Vil2*^*kd/kd*^ mice Suppl Fig. S3. Therefore, proximal tubular albumin reabsorption might be decreased in *Vil2*^*kd/kd*^ mice, although no significant change in the urinary albumin excretion rate was observed at the basal condition. These data support that the glomerular filtration barrier is not impaired in *Vil2*^*kd/kd*^ mice at the basal condition. Since a slight increase in RhoA activity and decrease in Rac1 activity were observed in *Vil2*^*kd/kd*^ mice at the basal condition, the filtration barrier might be tightly formed. The functional roles of ezrin in proximal tubular protein reabsorption thus require further examination.

Furthermore, Hugo *et al*. reported increased intensity of ezrin positive staining in several experimental glomerular disease rat models, including passive Heyman nephritis, aminonucleoside nephrosis, anti-GBM nephritis and 5/6 nephrectomy^23^. They reported that ezrin expression levels were markedly increased in mitotic podocytes. Their results support that loss of ezrin prevents the morphological changes in podocytes due to glomerular injury observed in our study. On the contrary, Wasik *et al*. reported that ezrin was down-regulated in diabetic glomeruli, although expression levels of podocalyxin and NHERF2 were also decreased^24^. In diabetic glomerulopathy, reduction of podocalyxin might be associated with podocyte injury. Further studies are required to clarify the precise role of ezrin in the progression of these diseases.

In summary, our study revealed that ezrin is not necessary for the maintenance of glomerular foot process integrity at the basal condition, but it does play an important role in the regulation of actin dynamics in glomerular podocytes through the regulation of Rac1 signalling. Ezrin seems to be associated with Rac1 activation when glomerular podocytes are injured, and the suppression of ezrin expression might thus be beneficial for protection of proteinuria in glomerulopathies.

## Methods

### Animal study

This study used 8- to 11-week-old male WT and *Vil2*^*kd/kd*^ mice. *Vil2*^*kd/kd*^ mice were generated as previously reported and were hybrids (129SV and C57BL6/J)^3^. All animals were maintained in metabolic cages fed a standard diet and tap water for 1 week, with daily measurements of their urine volume, water consumption and food intake. After spot urine collection, blood and kidneys were collected on the day of sacrifice. Plasma and urinary creatinine were measured by FujiDRI-Chem (Fujifilm, Tokyo, Japan). Urinary albumin was measured by enzyme immunoassay (TaKaRa Bio, Osaka, Japan). To investigate the influence of glomerular injury, a single dose of ADR (50 mg/kg body weight; Wako Purechemical Inc., Tokyo, Japan) was injected intravenously. Seven days after ADR injection, mice were sacrificed and their kidneys, blood and spot urine were collected. Alternatively, 200 μg of LPS (*Escherichia coli* O111:B4 strain, Invivogen, San Diego, CA) was injected intraperitoneally. The mice were sacrificed 24 h after injection, and kidneys, blood and spot urine were collected. All experiments were approved by the committee on laboratory animal care and use at Ritsumeikan University (BKC2013-035, approved on April 4, 2014) and performed according to university guidelines.

### Antibodies

Monoclonal anti-ezrin antibody was purchased from LifeSpan Bioscience Inc. (Seatle, WA). Polyclonal anti-ezrin, ERM, phospho-ERM, β-actin and GAPDH antibodies were purchased from Cell Signaling (Danvers, MA). Goat anti-mouse podocalyxin antibody was purchased from R&D Systems (Minneapolis, MN). Polyclonal anti-Rho-GDIα antibodies were purchased from Sigma Aldrich (St. Louis, MO). Polyclonal anti-radixin, moesin, podocin and synaptopodin antibodies were purchased from Abcam (Cambridge, MA). Polyclonal anti-NHERF2 antibody was purchased from Thermo Fischer Scientific (Waltham, MA). Polyclonal anti-CLIC5 antibody was purchased from Alomone Labs (Jerusalem, Israel). For immunoblotting, all antibodies were diluted with Solution 1 (Can Get Signal; TOYOBO, Osaka, Japan) by a factor of 1:1000. For immunofluorescence analysis, all antibodies were diluted with Solution A (Can Get Signal; TOYOBO, Osaka, Japan) by a factor of 1:100.

### Histological analysis and imaging for renal glomeruli by immunostaining

H & E staining, PAS staining, immunofluorescence analysis and electron microscopy were performed as previously reported^2^. Briefly, mouse kidneys were fixed with 4% paraformaldehyde (PFA) in 0.1 M phosphate buffer (PB) (pH 7.4), embedded in paraffin for histochemistry, and then sectioned (4 μm). After deparaffinisation and rehydration, the sections were used for haematoxylin and eosin. For immunofluorescence analyses, sections were immersed in a 0.01 M citrate buffer and autoclaved to activate immunogenicity after rehydration. Sections were then washed in phosphate-buffered saline (PBS) and incubated with primary antibodies overnight at 4 °C. Next, sections were washed with PBS-T (0.1% tween 20 in PBS) and then incubated with the secondary antibodies for 1 h at room temperature. After a final wash with PBS-T, the sections were mounted with a fluorescent mounting medium (VECTASHIELD, Vector Laboratories Inc., CA) and examined using a confocal laser-scanning microscope (FV-1000D IX-81, Olympus, Tokyo, Japan). For electron microscopy, mouse kidneys were fixed with 2% PFA and 2% glutaraldehyde (GA) in 0.1 M PB (pH 7.4) at 4 °C overnight. After fixation, tissues were washed three times with 0.1 M PB for 30 min, followed by post fixation with 2% osmium tetroxide (OsO_4_) in 0.1 M PB at 4 °C. Tissues were dehydrated using a series of graded ethanol (50%, 70%, 90% and 100%), infiltrated with propylene oxide (PO), and put into a 70:30 mixture of PO and resin (Quetol-812) for 1 h. The samples were then transferred to fresh 100% resin, which was polymerised at 60 °C for 48 h. Ultra-thin sections (70 nm) were prepared using an ultramicrotome (ULTRACUT UCT; Leica Biosystems, Germany). The sections were stained with 2% uranyl acetate and lead stain solution (Sigma Aldrich Japan, Tokyo, Japan). The sections were then examined using a transmission electron microscope (JEM-1200EX, JEOL Ltd., Tokyo, Japan) and digital images were taken with a CCD camera.

### Isolation of glomeruli from WT and *Vil2*^*kd/kd*^ mice

Mouse glomeruli were isolated by modification of Takemoto’s method^15^. Briefly, type IV Collagenase, Dynabeads M-450 Tosylactivated (Veritas Corp., Tokyo, Japan), a magnetic particle concentrator, and cell strainers (70 μm and 100 μm; BD Biosciences, CA) were used. Mice were anaesthetised with pentobarbital. The kidneys were perfused after ligation of the distal abdominal aorta, distal inferior cava vein, superior mesenteric, coeliac arteries and proximal abdominal aorta. The inferior cava vein was cut for venous drainage after the insertion of a polyethylene tube with a winged needle into the middle of the abdominal aorta. Kidneys were perfused with 20 mL of ice-cold PBS and 20 mL of Dynabeads at a concentration of 2 × 10^6^ beads/mL PBS. The kidneys were then removed, minced and digested with collagenase (1 mg/mL PBS) at 37 °C for 30 min. After digestion, tissue was gently passed through a 100-μm cell strainer, followed by an ice-cold PBS flush. The cell suspension was filtered using a 70-μm cell strainer. Glomeruli that contained Dynabeads were isolated by a magnetic particle concentrator and washed three times with ice-cold PBS.

### Immunoblot analysis of protein expression levels in isolated mouse glomeruli

Immunoblot analysis was performed as previously reported^2,25^. Isolated glomeruli were lysed by lysis buffer composed of 150 mM NaCl, 3 mM KCl, 5 mM EDTA, 3 mM ethylene glycol tetraacetic acid (EGTA) and 50 mM Tris-HCl (pH 7.4) containing 1% Triton X-100. After centrifugation at 3,000 × g at 4 °C for 10 min to remove cell debris and magnetic beads, the supernatants were carefully collected. 5–10 μg of protein was loaded into each lane for Laemmli SDS-polyacrylamide gel electrophoresis (8–12.5%), and then transferred to a polyvinyliden difluoride membrane. The membrane was blocked for 1 h using 2.5% milk powder in TBST (10 mM Tris-HCl, pH 8.5, 150 mM NaCl and 0.1% Tween 20), and exposed to primary antibodies diluted with Solution 1 (Can Get Signal; TOYOBO, Osaka, Japan) overnight at 4 °C. After a TBST rinse, the secondary antibody diluted with Solution 2 (Can Get Signal; TOYOBO, Osaka, Japan) was applied to the membrane for 1 h at room temperature. After washing, antigen–antibody complexes were visualised with a chemiluminescence system (Immobilon, Merck Millipore, Darmstadt, Germany).

### Measurement of rho activity in isolated glomeruli

The activity of RhoA, Rac1 and Cdc42 in isolated glomeruli was measured by a G-LISA activation assay following the manufacturer’s instructions (Cytoskeleton, Inc., Denver, CO). Briefly, isolated glomeruli were immediately lysed using the ice-cold cell lysis buffer provided with the G-LISA kit, and magnetic beads and cell debris were removed by centrifugation at 3,000 × g for 15 min at 4 °C. Equal amounts of proteins (10 to 50 μg) were applied to the Rho-GTP affinity G-LISA plate and incubated for 30 min at room temperature. The active GTP-bound forms of RhoA, Rac1 and Cdc42 were measured using indirect immunodetection, followed by a colorimetric reaction at 490 nm on a microplate spectrophotometer.

### Immunofluorescence analysis of E11 cells transiently transfected with ezrin mutants

E11 cells transiently transfected with ezrin constructs were washed with ice-cold PBS and fixed by PFA for 30 min. Next, cells were washed with ice-cold PBS containing 20-mM glycine and permeabilised by PBS containing 1% Triton-X100. After washing with PBS, cells were stained by rhodamine-phalloidin and incubated with the Flag M2 antibody (1:100) at 4 °C overnight. Cells were washed with PBS containing 1% Tween and stained by the Alexa 488 conjugated anti-mouse antibody (Invitrogen). Cells were then observed using confocal laser microscopy FV10i (Olympus). More than 20 clones were examined and the number of lamellipodia per cell was counted. Lamellipodia were defined as 3- to 10-μm regions located at the cell periphery, as previously described by Raucher *et al*.^26^.

### Cell culture and *in vitro* experiments using a podocyte cell line

E11 cells, a conditionally immortalised mouse podocyte cell line, were purchased from Cell Lines Service (Eppelheim, Germany). Cells were kept in RPMI1640 supplemented with 10% foetal bovine serum, 100 units/mL penicillin and 100 μg/mL streptomycin. Cells were cultured at 33 °C for propagation and at 37 °C for differentiation for 14 days. Negative control siRNA (Ambion: #4390843) and ezrin siRNA (Ambion: #4390771) were prepared, and transfection of siRNA was performed using lipofectamine RNAimax (Thermo Fischer Scientific, MA). Four days after treatment of siRNA, cell surface biotinylation was performed as previously reported^2^. Briefly, E11 cells were biotinylated with sulfosuccinimidyl-2-(biotinamido)ethyl-1,3-dithiopropionate (sulfo-NHS-SS-Biotin). Cells were then rinsed with ice-cold PBS containing 0.1 mM CaCl_2_ and 1 mM MgCl_2_ and incubated with 1 mg/mL EZ-Link Sulfo-NHS-SS-biotin (Thermo Fischer Scientific) in PBS (pH adjusted to 8.0) for 30 min. Cells were rinsed with cold PBS, and unreacted biotin molecules were quenched with PBS containing 1% BSA for 10 min before cell lysis and streptavidin-agarose capture. The captured proteins were eluted by the SDS-sample buffer and used for the immunoblot analysis. On the other hand, pcDNA3.1(-)-N-terminal flag-tagged ezrin and other mutants (T567A, T567D and N-terminal (1-310), ΔC-terminal (1-533)) were transfected to E11 cells by lipofectamine 2000 (Thermo Fisher Scientific, Kanagawa Japan). Two days after transfection, cells were collected for analysis of Rho-GTPase activity and immunofluorescence analysis.

### Statistical analysis

Statistical analysis was performed using a student *t*-test or one-way ANOVA with Turkey’s *post-hoc* test in GraphPad Prism 6.0 (San Diego, CA). A *P*-value of <0.05 was considered statistically significant.

## Electronic supplementary material

Supplementary information
