# Supplementary material for: Loss of ezrin expression reduced the susceptibility to the glomerular injury in mice
Source: Sci Rep. 2018 Mar 14;8:4512. doi: 10.1038/s41598-018-22846-0 (PMC5852236; doi:10.1038/s41598-018-22846-0)

Supplementary Information

Loss of ezrin expression reduced the susceptibility to the glomerular injury in mice

Ryo Hatano[1*], Ai Takeda[1], Yukiko Abe[1], Kotoku Kawaguchi[1], Itsuro Kazama[2], Mitsunobu Matsubara[3], Shinji Asano[1]

[1]Department of Molecular Physiology, College of Pharmaceutical Sciences, Ritsumeikan University, Kusatsu, Shiga, Japan [2]Department of Physiology, Tohoku University Graduate School of Medicine, Sendai, Miyagi Japan [3]Division of Molecular Medicine, Center for Translational and Advanced Animal Research, Tohoku University Graduate School of Medicine

Supplementary Materials

Figure Legends

Figure S1. Immunofluorescent analysis for ERM proteins in glomeruli.

Coimmunofluorescent analysis for ezrin (a) and moesin (b) with CD34, an endothelial cells marker, and podocalyxin, a podocyte marker were performed. Moesin was coimmunostained with desmin, a mesangial marker (c). But no apparent colocalization between moesin and desmin was observed. Localizations of radixin and moesin were also investigated in (d) WT and (e) *Vil2*$^{kd/kd}$ mouse glomeruli. Ezrin, radixin, and moesin were coimmunostained with podocalyxin.

Figure S2. Detection of phosphorylated ERM proteins in the glomeruli after ADR injection.

Glomeruli were isolated from WT and *Vil2*$^{kd/kd}$ mice with saline injection or ADR injection. Total ERM protein and phosphorylated ERM proteins were investigated by immunoblotting using ERM antibody and phospho-ERM antibody using glomeruli from ADR-injected (a) and LPS-injected mice (b). The ratio of phosphorylated ezrin versus total ezrin was calculated (ADR: n = 6, each, LPS: n =3, each).

Figure S3. Urinary β2-microglobulin was measured by ELISA

Urinary β2-microglobulin was measured by ELISA (USCN Lifescience Inc., China). Spot urine was collected from WT and Vil2kd/kd mice. Data was normalized by the urinary creatinine (n = 10, each).

Figure S4. Full-length images of the cropped gels presented the main figures.

a) Full-length image of Figure 1f
b) Full-length image of Figure 4a
c) Full-length image of Figure 4d

Figure S5. Full-length images of the cropped blots presented the main figures.

a) Full-length image of Figure 3a (ezrin)
b) Full-length image of Figure 3a (β-actin)
c) Full-length image of Figure 3a (NHERF2)
d) Full-length image of Figure 3a (podocalyxin)
e) Full-length image of Figure 3a (RhoGDIα)
f) Full-length image of Figure 3a (CLIC5)
g) Full-length image of Figure 3a and supplemental Figure S2 (ERM)
h) Full-length image of Supplemental Figure S2 (phospho-ERM)

Figure S6. Full-length images of the cropped blots presented the main figures.

a) Full-length image of Figure 6a (ezrin)
b) Full-length image of Figure 6a (β-actin)
c) Full-length image of Figure 6b (podocalyxin)
d) Full-length image of Figure 6b (β-actin)

Supplemental Figure S1

a)

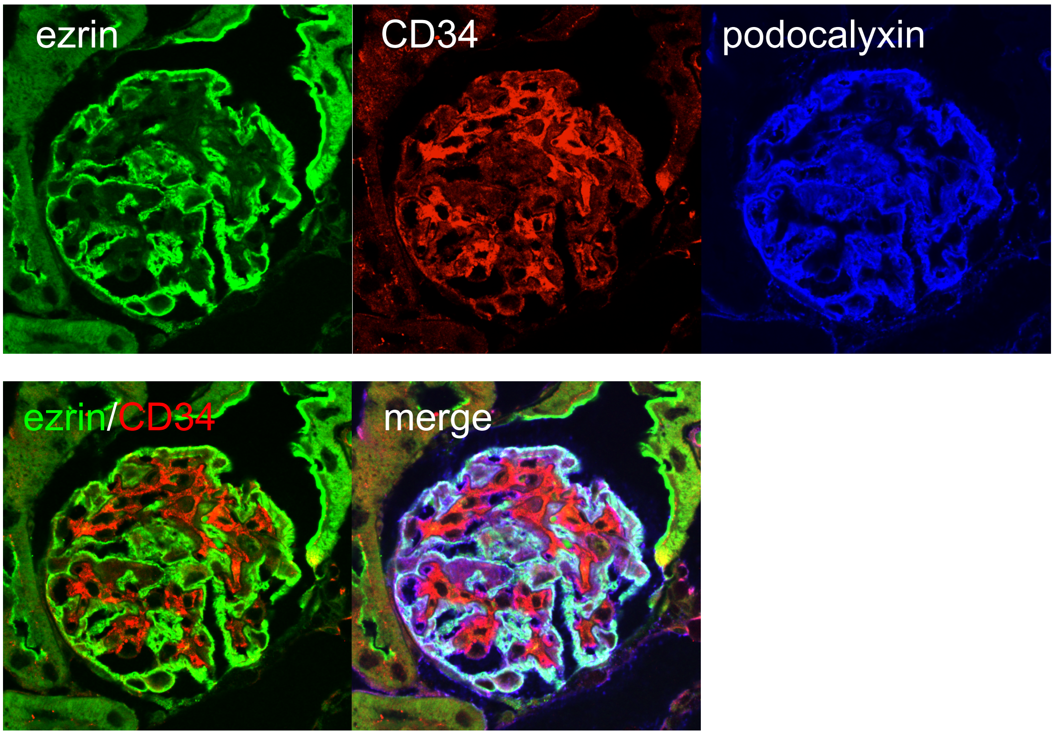

b)

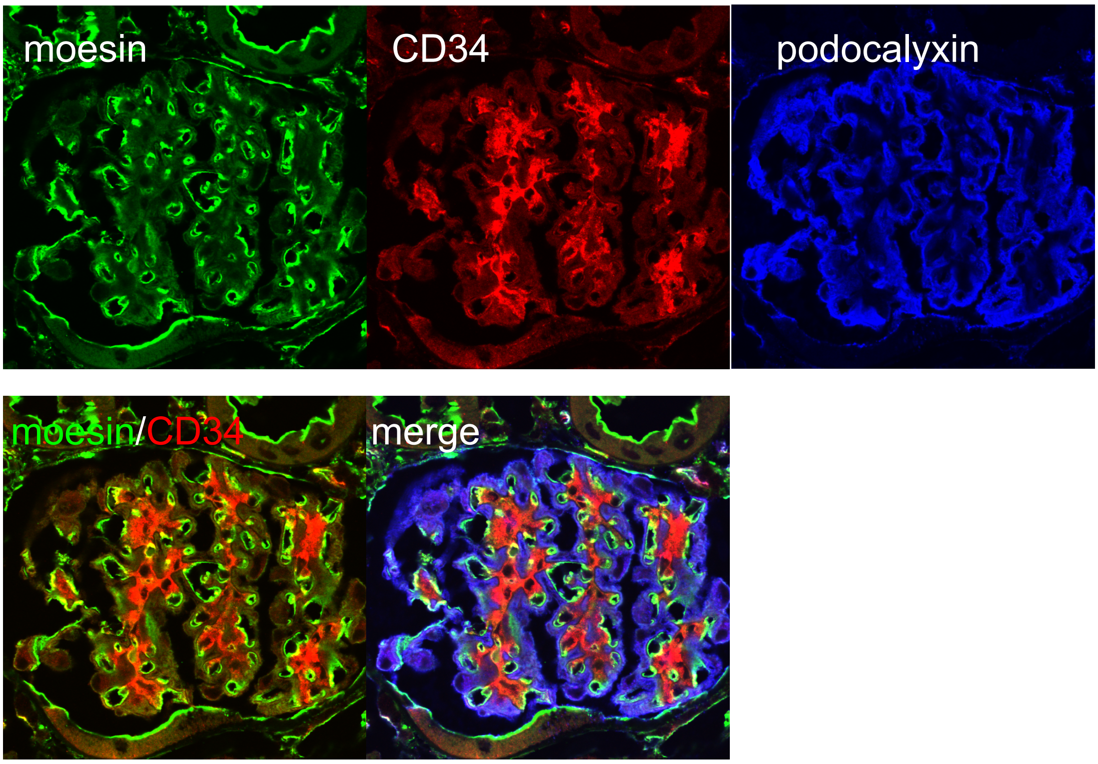

c)

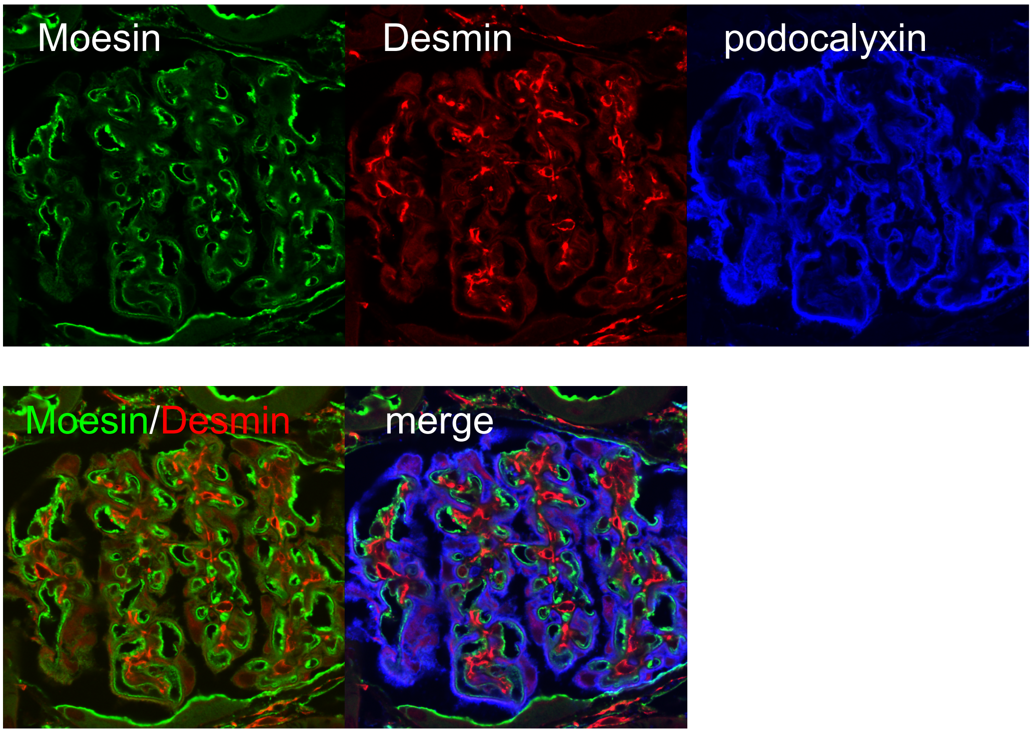
Moesin
Desmin
podocalyxin
Moesin/Desmin
merge

d)

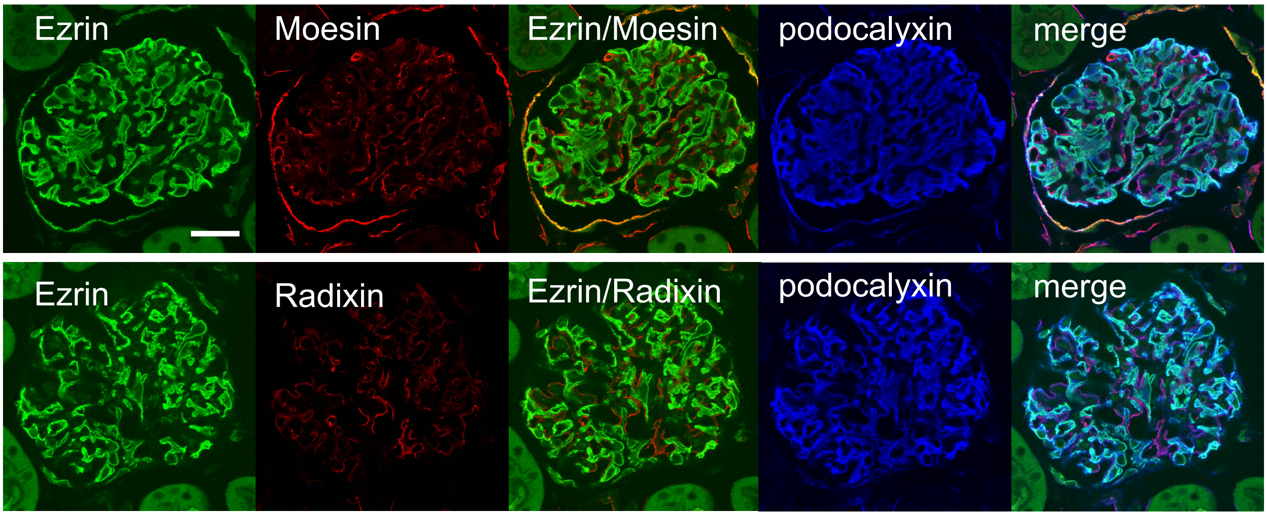
Ezrin
Moesin
Ezrin/Moesin
podocalyxin
merge
Ezrin
Radixin
Ezrin/Radixin
podocalyxin
merge

e)

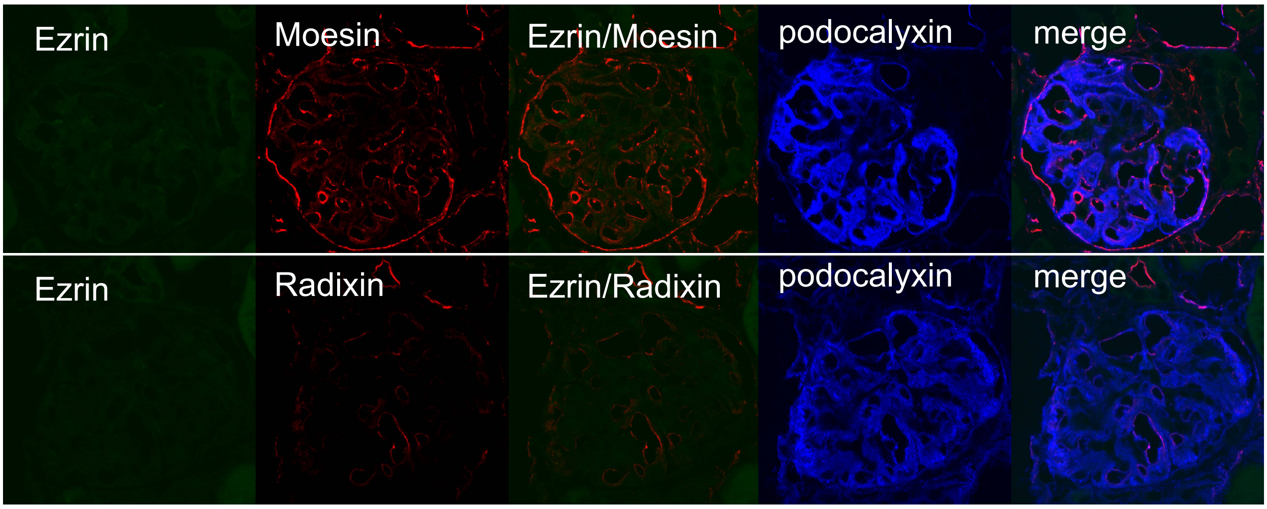
Ezrin
Moesin
Ezrin/Moesin
podocalyxin
merge
Ezrin
Radixin
Ezrin/Radixin
podocalyxin
merge

Supplemental Figure S2

a)

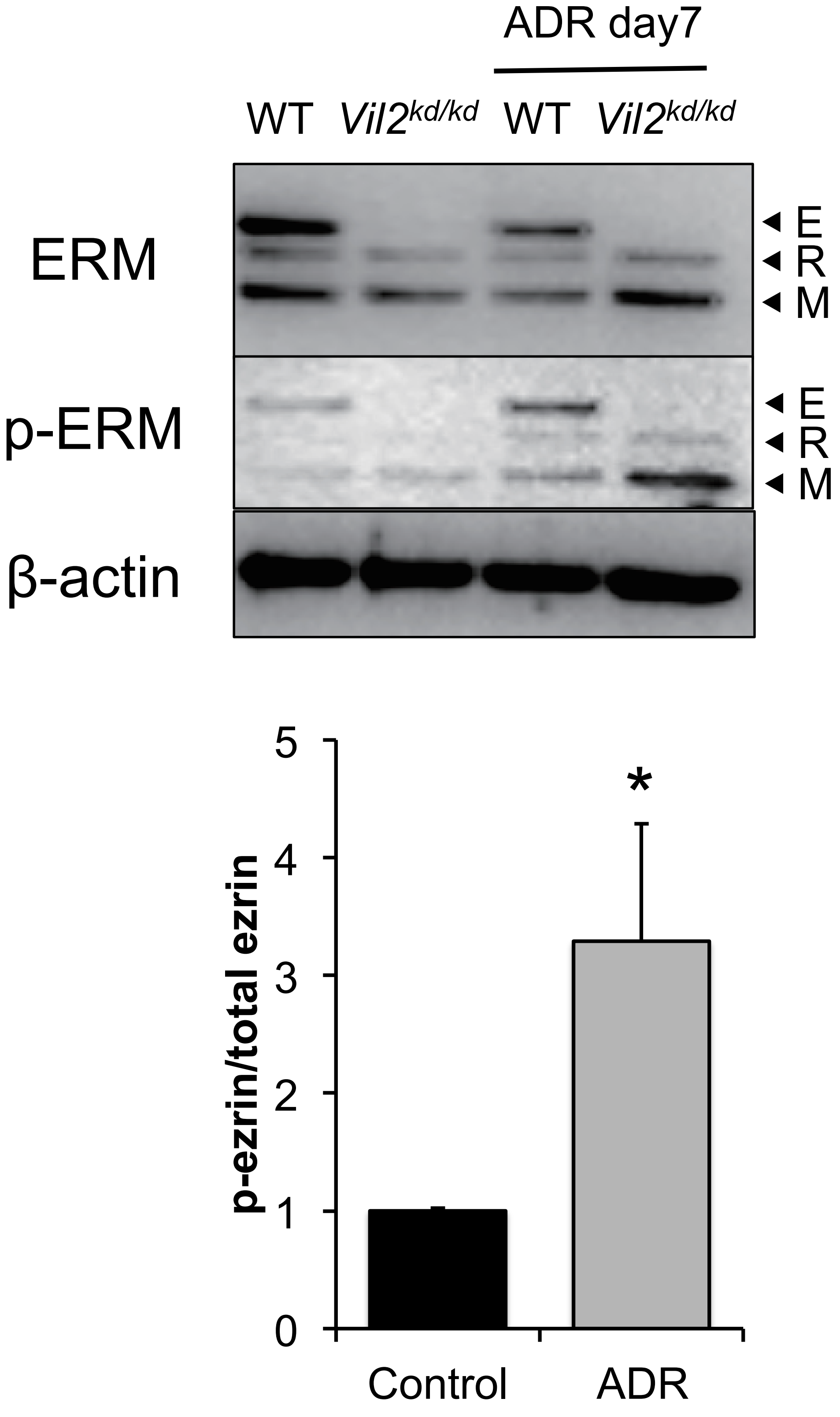

b)

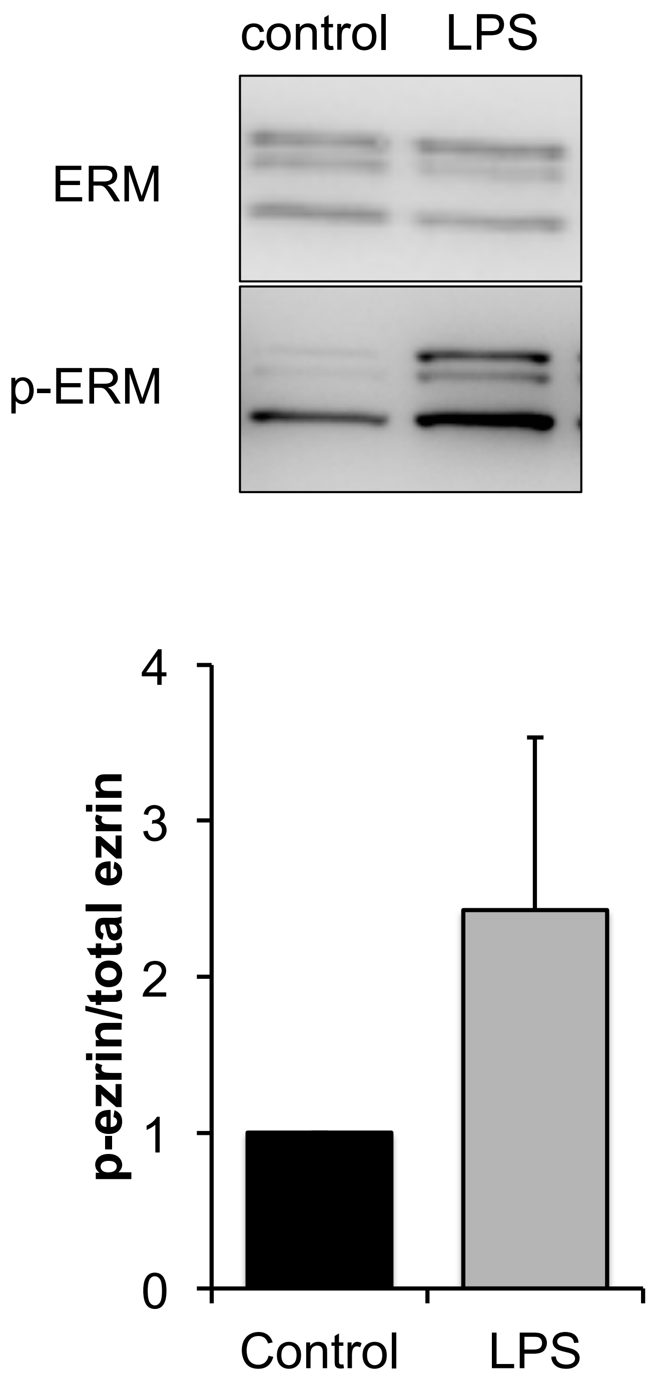

Supplemental Figure S3

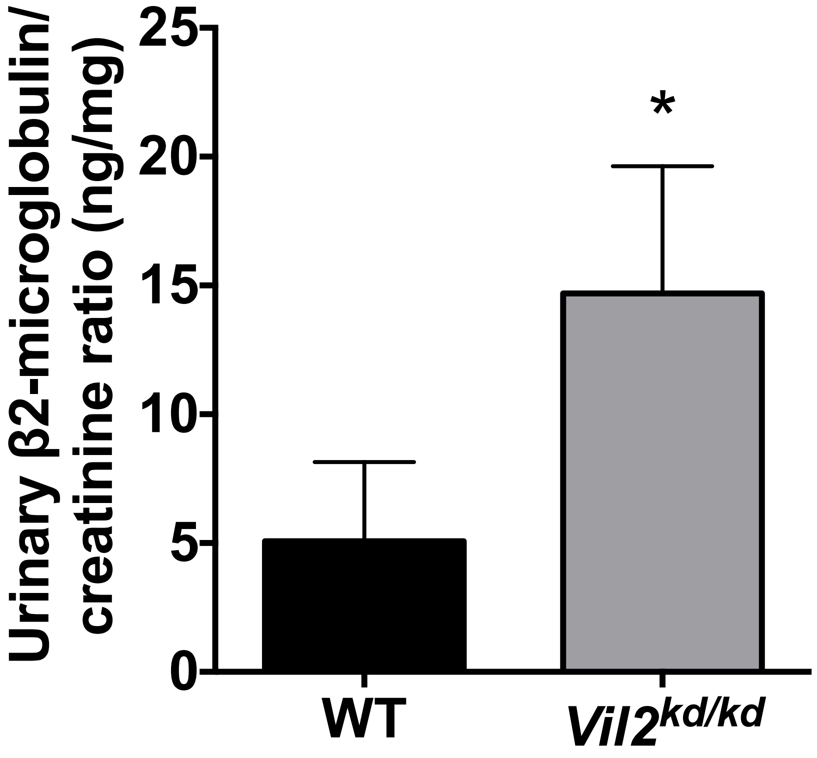

Supplemental Figure S3

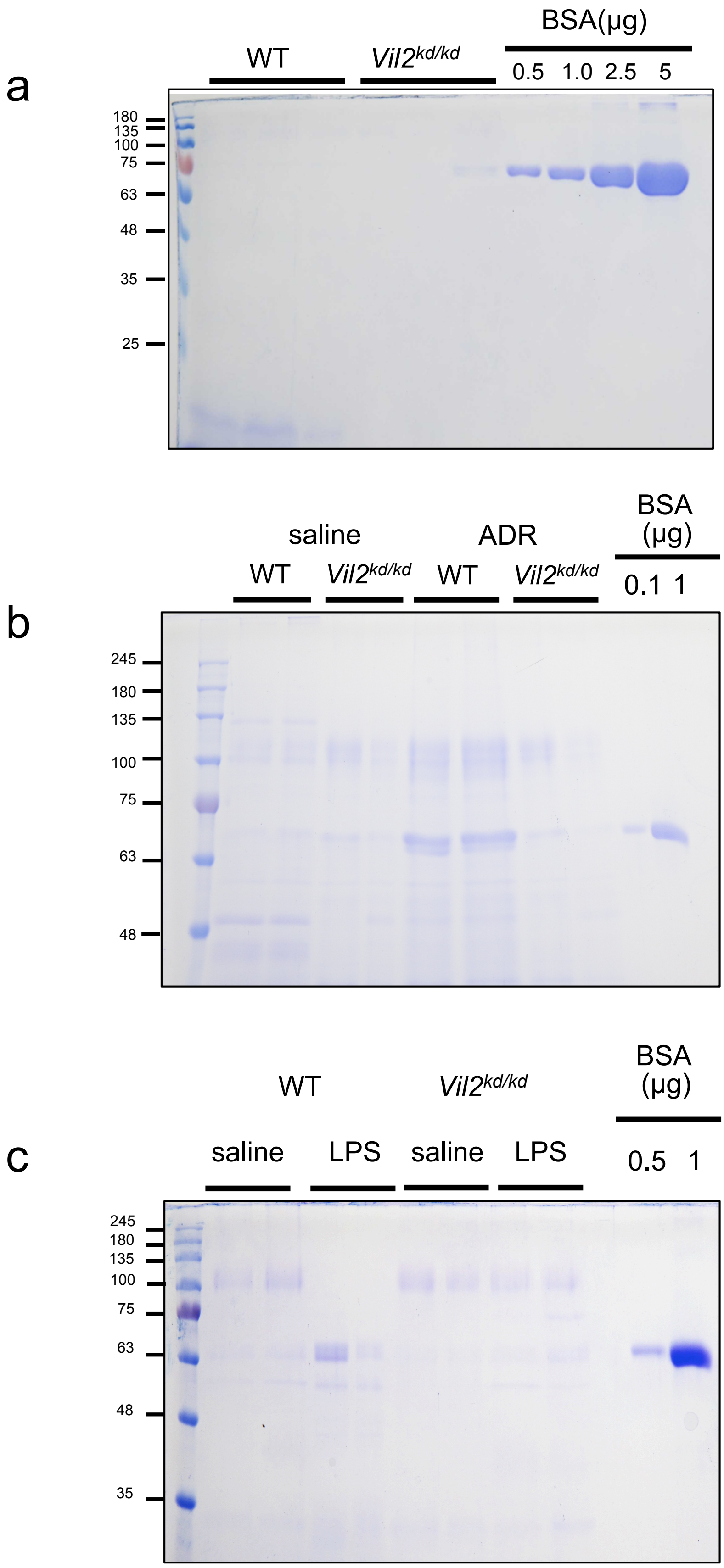

Supplemental Figure S4

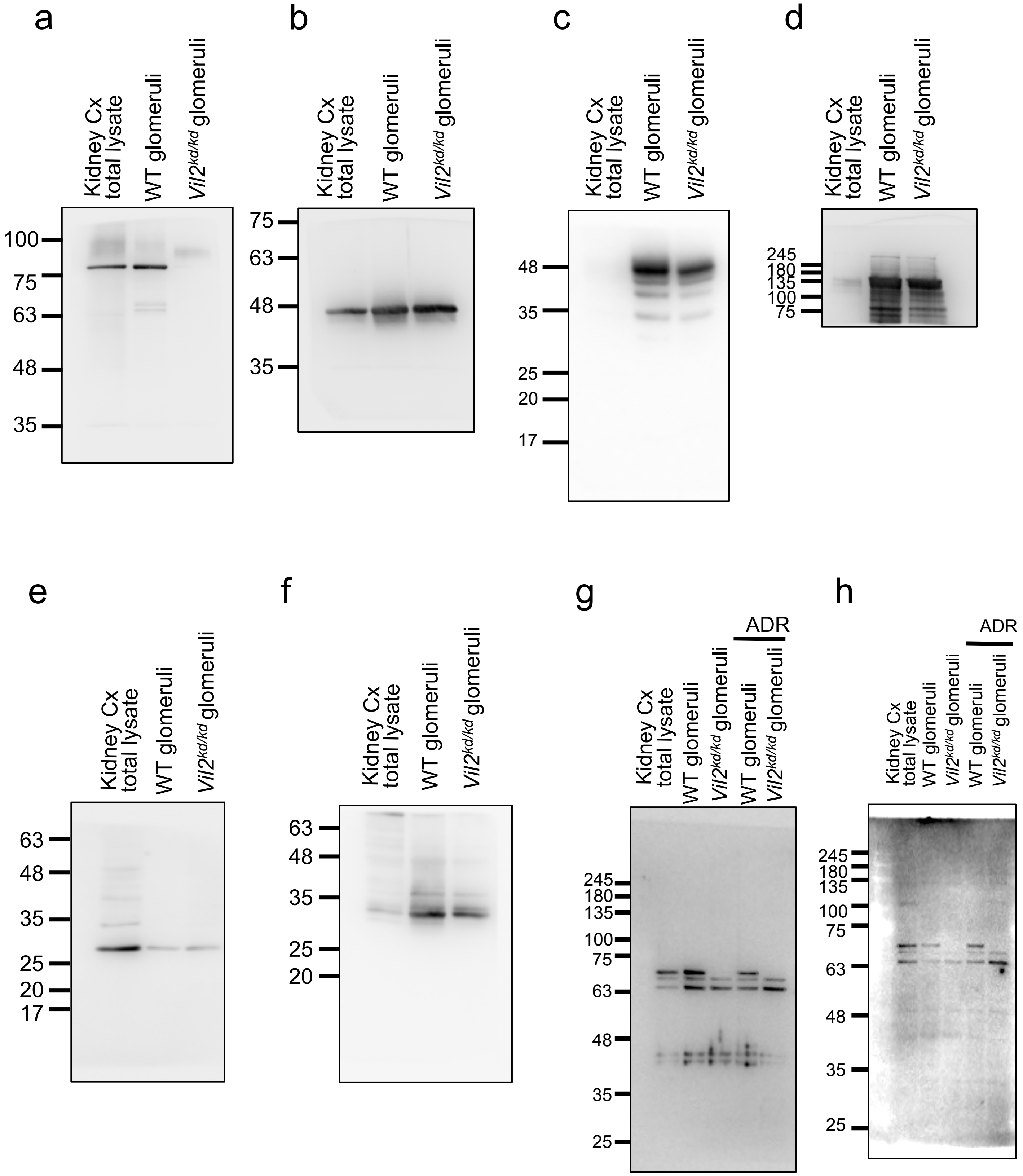

Supplemental Figure S5

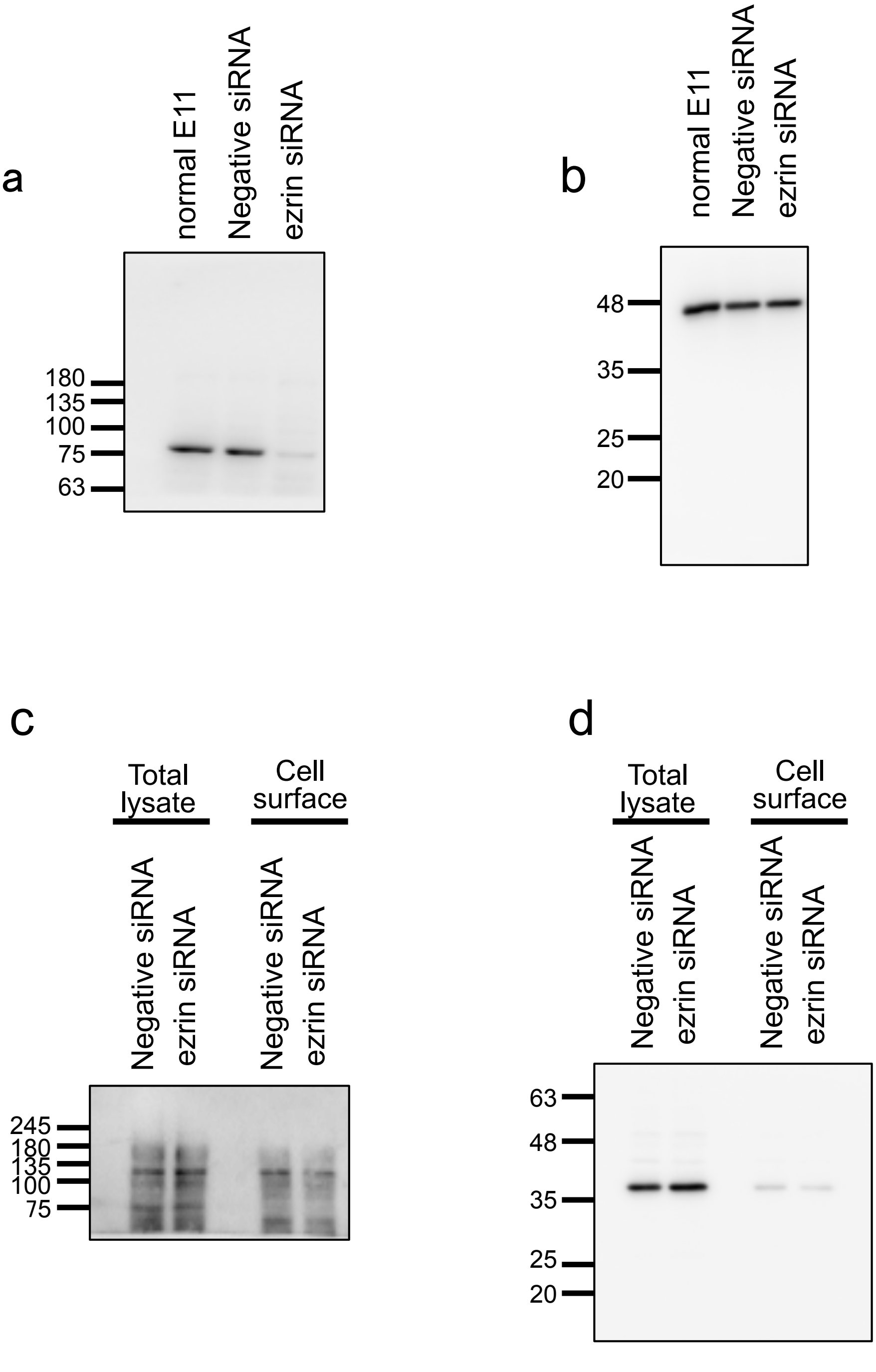

Supplement: Supplementary file 1 — Supplementary information [file 41598_2018_22846_MOESM1_ESM.pdf]
